# Transcriptome Analysis of Leaf Senescence Regulation Under Alkaline Stress in *Medicago truncatula*

**DOI:** 10.3389/fpls.2022.881456

**Published:** 2022-04-28

**Authors:** Shuwei Dong, Wenhui Pang, Zhe Liu, He Li, Kangning Zhang, Lili Cong, Guofeng Yang, Zeng-Yu Wang, Hongli Xie

**Affiliations:** Key Laboratory of National Forestry and Grassland Administration on Grassland Resources and Ecology in the Yellow River Delta, College of Grassland Science, Qingdao Agricultural University, Qingdao, China

**Keywords:** alkaline stress, *Medicago truncatula*, leaf senescence, transcriptome analysis, senescence-associated genes (SAGs)

## Abstract

In plants, the leaf is an essential photosynthetic organ, and is the primary harvest in forage crops such as alfalfa (*Medicago sativa*). Premature leaf senescence caused by environmental stress can result in significant yield loss and quality reduction. Therefore, the stay-green trait is important for improving the economic value of forage crops. Alkaline stress can severely damage leaf cells and, consequently, cause leaf senescence. To understand the molecular regulatory mechanisms and identify vital senescence-associated genes under alkaline stress, we used high-throughput sequencing to study transcriptional changes in *Medicago truncatula*, a model plant for forage crops. We identified 2,165 differentially expressed genes, 985 of which were identical to those in the dark-induced leaf senescence group. Gene ontology (GO) and Kyoto Encyclopedia of Genes and Genomes (KEGG) pathway enrichment analyses showed that the 985 genes were mainly enriched in nutrient cycling processes such as cellular amino acid metabolic processes and organic substance catabolic processes, indicating nutrient redistribution. The other 1,180 differentially expressed genes were significantly enriched in the oxidoreductase complex, aerobic respiration, and ion transport. Our analysis showed the two gene sets guiding the coupled physiological and biochemical alterations play different roles under alkaline stress with a coordinated and integrated way. Many transcription factor families were identified from these differentially expressed genes, including *MYB*, *WRKY*, *bHLH*, and *NAC* which have particular preference involved in stress resistance and regulation of senescence. Our results contribute to the exploration of the molecular regulatory mechanisms of leaf senescence in *M. truncatula* under alkaline stress and provide new candidate genes for future breeding to improve the biomass and quality of forage crops.

## Introduction

Senescence occurs when the photosynthetic efficiency in a leaf is constantly decreasing; its stages include a color change from green to yellow, wilting, and death. During senescence, the nutrients in the leaf are transferred to new buds, developing flowers, maturing seeds, or other plant development, thus directly enhancing plant adaptability and reproductive success in the face of stress (Uauy et al., 2006; Lim et al., 2007). Therefore, the timing of leaf senescence is of biological significance. Premature senescence caused by environmental stressors can result in significant yield loss and quality reduction. Such stressors include darkness, drought, saline, and alkaline conditions (Guo and Gan, 2012; He et al., 2018; Guo et al., 2021). Saline-alkaline stress is a common abiotic stress that limits plant growth and development, and has become a serious problem restricting crop production as well as ecological environment construction (Zhu, 2016; Wei et al., 2021). High salinity accelerates leaf senescence, thereby reducing plant biomass (Balazadeh et al., 2010; Allu et al., 2014; Yang and Guo, 2018b). We confirmed this phenomenon in our previous study, and preliminarily investigated the associated molecular mechanisms (Dong et al., 2021).

Unlike salt stress, alkaline stress is caused mainly by NaHCO_3_ and Na_2_CO_3_. Therefore, Na^+^ stress occurs in alkali stress, as well as HCO_3_^–^ and pH stresses (Zhang et al., 2019). It can induce ion toxicity, osmotic stress, and oxidative damage in plants leading to accelerated leaf senescence (Fan et al., 2021). Alkaline stress can significantly disrupt ion balance and interfere with the uptake of mineral elements, resulting in excessive Na^+^ accumulation in leaf cytoplasm, thereby producing ion toxicity and inducing leaf senescence (Ghanem et al., 2008; Guo et al., 2009; Yang and Guo, 2018b). The decreasing K^+^/Na^+^ ratio disrupts the ultrastructure of chloroplasts, leading to chlorophyll degradation, a reduced photosynthetic rate, and accelerated leaf senescence (Zhao et al., 2001; Wang et al., 2019). Excess ions produce osmotic stress and lead to dehydration in leaves, followed by rapid leaf senescence (Yang and Guo, 2018a; Zhang P. et al., 2021).

The effect of oxidative damage on leaf senescence requires investigation. High pH stress results in increased permeability of the cell membrane in leaves by inducing the accumulation of malondialdehyde (MDA) and reactive oxygen species (ROS), allowing penetration by small molecules of organic substances and electrolytes into the cell. The intracellular molecular structures and functions are in turn damaged, accelerating leaf senescence (An et al., 2016; Zou et al., 2020). Plants have developed a series of regulatory adaptive mechanisms to resist senescence, such as alleviation of osmotic stress, modulation of ion homeostasis, and antioxidant protection (Yang and Guo, 2018b; Wei et al., 2020). It has been reported that with leaf senescence, numerous leaf senescence-associated genes (SAGs) are expressed and associated transcription factors (TFs) are involved in regulation (Buchanan-Wollaston, 1997; Guo and Gan, 2005). TF families (such as NAC, MYB, WRKY, and bZIP) have been shown to participate, often critically, in the regulation of senescence in plants (Hao et al., 2010; Mao et al., 2017; Woo et al., 2019; Xu et al., 2020; Dong et al., 2021). During leaf senescence, a large number of SAGs and TFs are expressed at high levels, and these genes constitute several complex senescence regulatory networks that are interlinked and regulated by each other to control leaf senescence. However, there is little understanding of the relationship between SAGs and alkaline stress.

In recent years, significant progress has been made in elucidating the relationship between SAGs and abiotic stresses in *Arabidopsis thaliana* (Breeze et al., 2011), tobacco (Pageau et al., 2006) and rice (Lee et al., 2001). At present, few reports have been published on the mechanism of leaf senescence in leguminous forage species (Chao et al., 2018; Yuan et al., 2020). However, the key regulators of leaf senescence induced by alkaline stress remain unclear. Alfalfa (*Medicago sativa* L.) is considered to be one of the most important forages in the world because of its high yield, high quality, and wide range of adaptations (Bouton, 2007; Wang et al., 2016). Most of nutrients in alfalfa are stored in the leaves, and leaf senescence can greatly affect the nutritional quality of the plant, especially when affected by environmental factors such as saline and alkaline stress. Therefore, preventing premature senescence or delaying senescence appropriately to increase biomass accumulation is important for improving alfalfa quality and increasing agricultural economic efficiency (Zhou et al., 2011). *Medicago truncatula* has been adopted as a suitable model for studying forage crop improvements and leaf senescence (Barker et al., 1990; Zhang et al., 2014). The highly controlled repeatable detached leaves are widely used to evaluate leaf senescence in different plant species (Mao et al., 2017; He et al., 2018; Sakuraba et al., 2018).

In a previous study, we investigated salt- and dark-induced leaf senescence in *M. truncatula* by collecting transcriptional data over the course of leaf senescence. In this study, we investigated the relationship between leaf senescence and alkaline stress by analyzing detailed expression profiles and annotating the SAGs. The purpose of this study was to identify the genes involved in alkali-induced leaf senescence so as to provide new candidate genes for breeding management strategies.

## Materials and Methods

### Plant Material and Alkaline Stress Treatments

The *M. truncatula* ecotype R108 was used in this study. Seeds that had already been vernalized for 2 days were sown in dishes with moistened filter paper and grown in a light incubator for 7 days. They were then transferred into Hoagland’s nutrient solution for hydroponic growth cultivation, and the culture medium was changed every 3 days. Plants were placed in a light incubator with a 16 h photoperiod, day/night temperatures of 25°C/22°C, and a relative humidity of 60–70%.

After 5 weeks, the third compound leaf of each plant was removed and immediately transferred into Petri dishes containing 0, 10, 20, and 40 mM NaHCO_3_ solution [prepared with half Murashige–Skoog medium, 3 mM MES (2-morpholine ethyl sulfonic acid) buffer, adjusted to pH 5.8]. The Petri dishes were then placed under light or dark conditions, with the growth conditions: 16 h light (25)/8 h darkness (22°C), relative humidity of 60–70%, and light intensity of 300 mol/m^2^⋅s.

Individual samples were harvested at 0, 2, 4, and 6 days post-alkaline salt stress treatment and briefly immersed in liquid nitrogen before being stored at −80°C. The sampled materials were used to measure physiological indicators [chlorophyll, MDA, H_2_O_2_, and abscisic acid (ABA)] and for transcriptomic sequencing.

Three biological replicates were analyzed for each sample group. All data were subjected to one-way analysis of variance (ANOVA) using SPSS 26 (IBM Corp., Armonk, NY, United States). Mean differences were analyzed using Duncan’s multiple range test, and statistical significance was set at *P* < 0.05. All charts were created using Microsoft Excel 2019 (Microsoft Corp., Redmond, WA, United States).

A dark treatment group (dark) was established as a positive control to better screen the SAGs (Sobieszczuk-Nowicka et al., 2018). In addition, a light control group (control-light) was established to remove background effects, so as to acquire SAGs involved in senescence upon alkaline stress, not just the genes reacting to alkaline stress.

### RNA Quantification and Qualification

Total RNA extraction and quality control were conducted as per the method in an earlier study (Dong et al., 2021). Only high-quality RNA samples (OD_260/280_ = 1.8–2.2, ≥ 50 ng/μL, > 1 μg) were used for sequence library constructions.

### Library Preparation and Transcriptome Sequencing

RNA libraries were prepared using the TruSeq™ RNA sample preparation kit from Illumina (San Diego, CA, United States) using 1 μg of RNA. Messenger RNA (mRNA) was enriched and randomly fragmented into small fragments of approximately 200 bp, and cDNA synthesized using a SuperScript double-stranded cDNA synthesis kit (Invitrogen, CA, United States). The synthesized cDNAs were subjected to end-repair, phosphorylation, and “A” base addition according to Illumina’s library construction protocol. Libraries were size selected for 200–300 bp cDNA target fragments using 2% Low Range Ultra Agarose electrophoresis followed by enrichment of PCR (sample preparation kit; Illumina, San Diego, CA). After quantification using TBS380 (Turner BioSystems, Sunnyvale, CA, United States), the paired-end RNA-seq sequencing library was constructed on an Illumina HiSeq xten/NovaSeq 6000 platform, and 150 bp paired-end reads were generated.

Raw reads were trimmed, and their quality controlled by Fastp (Version: 0.19.5)^1^ to acquire clean reads. All downstream analyses were based on clean data.

All obtained high-quality and clean reads were separately aligned to the reference genome of *M. truncatula* (reference genome version MedtrA17_4.0; reference genome source can be accessed via http://plants.ensembl.org/Medicago_truncatula/Info/Index with orientation mode using hisat2 (Version 2.1.0)^2^ software. The mapped reads of each sample were assembled using StringTie (version 1.3.3 b).^3^

### Quantification of Gene and Differential Expression Analysis

StringTie was used to count the number of reads mapped to each gene. The transcripts per million reads (TPM) of each gene were calculated from gene length and the read count mapped to it. RNA-Seq by Expectation-Maximization (RSEM, Version 1.3.1)^4^ was applied to quantify gene abundance for each group and time point.

Differential expression analysis was performed using R statistical package software (EdgeR, Version 3.24.3).^5^ The resulting *P*-values were adjusted using Benjamini and Hochberg’s approach in order to control the false discovery rate. Genes with adjust < 0.05, |log_2_FC| ≥ 1 by EdgeR were defined as significantly different.

### Gene Ontology and Kyoto Encyclopedia of Genes and Genomes Pathway Enrichment Analysis of Differentially Expressed Genes

Gene Ontology (GO)^6^ functional enrichment was conducted using Goatools (Version 0.6.5)^7^ and Fisher’s precision tests. GO terms with BH-corrected P adjustment (<0.05) were considered significantly enriched by DEGs.

We used KOBAS (Version 2.1.1)^8^ and Fisher’s precision test for DEGs in the Kyoto Encyclopedia of Genes and Genomes (KEGG)^9^ pathways. The metabolic pathways were considered significantly enriched by DEGs at a BH-corrected value of *P* < 0.05.

### Transcription Factor Analysis

TFs are a class of proteins that bind to specific DNA sequences and are widely found in living organisms. They have an activating or blocking effect on gene expression. TF analysis was undertaken using PlantTFDB 4.0 (Version 4.0).^10^ A threshold of less than e^–5^ was used for the Hmmscan search.

### Time-Course Senescence-Associated Gene Analysis

Time-series SAG analysis based on the microarray Significant Profiles (maSigPro, Version 1.56.0)^11^ was performed to obtain genes with different expression profiles throughout the series of sampling time nodes.

A short time-series expression miner (STEM, Version 1.3.11) with a *P* < 0.05 threshold was used for temporal pattern analysis.

### Quantitative Real-Time PCR Analysis

Quantitative real-time PCR (qRT-PCR) was used to verify the reliability of RNA-seq data. The RNAs were reverse transcribed using the M5 Super Plus qPCR RT kit with gRNA remover (mei5 Biotech Co., Ltd., Beijing, China) and then subjected to qRT-PCR using ChamQ SYBR color qPCR Master Mix (Vazyme Biotech Co., Ltd., Nanjing, China). Three replicates were performed for each reaction. Twenty-five genes were chosen for qRT-PCR with the following criteria: TFs (Supplementary Table 5), 15 upregulated genes and 9 downregulated genes, belonging to profiles 21 and 4 of 985 SAGs, and differential expression in different groups. Mt UBC Q-2 served as the reference gene.

Gene-specific primers were designed using Primer 5.0 and are shown in Supplementary Table 1.

## Results

### Phenotypic and Physiological Responses of Detached Leaves to Alkaline Stress

**FIGURE 1:**
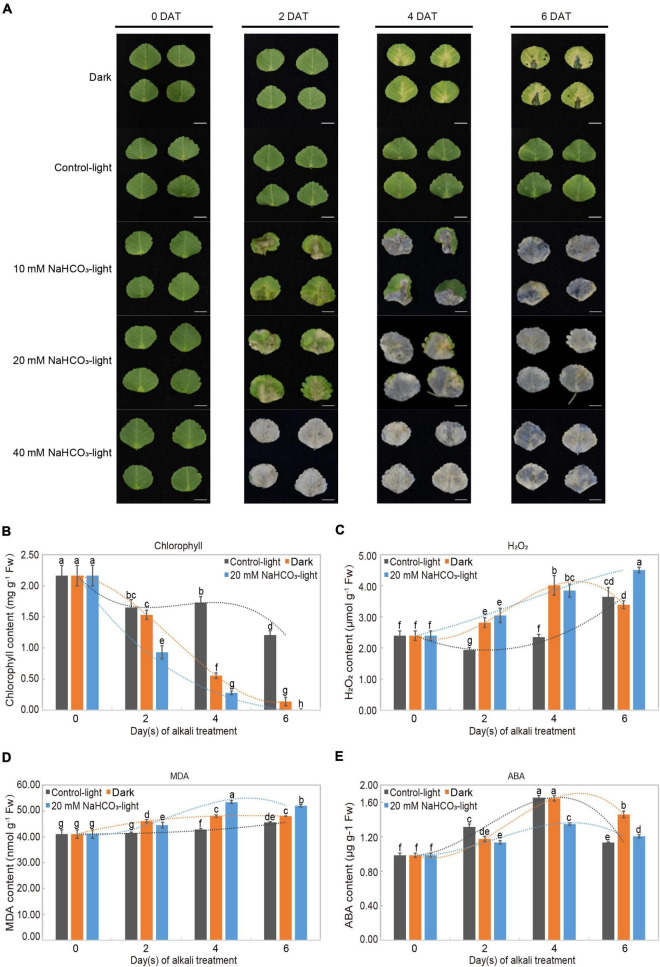
Alkali stress-induced leaf senescence and physiological analysis in *Medicago truncatula*. **(A)** Leaf senescence progression and color change of detached *M*. *truncatula* leaves under dark (Dark), normal light (Control-light), and 10, 20, 40 mM NaHCO_3_ (10 mM NaHCO_3_-light, 20 mM NaHCO_3_-light and 40 mM NaHCO_3_-light) conditions for 0, 2, 4, and 6 days. DAT: days after treatment. Scale bar = 1 cm. **(B)** Chlorophyll (a + b), **(C)** H_2_O_2_, **(D)** malondialdehyde (MDA), and **(E)** abscisic acid (ABA) in detached *M*. *truncatula* leaves exposed to different conditions during senescence. Values are presented as mean ± SE of three independent biological replicates per time point. Different letters indicate significant differences among treatments according to the analysis of variance (ANOVA, *P* < 0.05). Error bars correspond to standard error. FW, fresh weight.

The detached leaves showed different phenotypic changes across different groups (Figure 1A). In the control light group, leaves remained green throughout 6 days; in the dark group, leaves showed progressive yellowing from days 4 to 6; in the alkaline-stress groups, leaves treated with concentrations of 10 and 20 mM NaHCO_3_ turned yellow on day 2 and formed eroded lesions; over time, the leaves slowly turned transparent from necrosis. There was a significant correlation between the concentration of NaHCO_3_ and leaf phenotypic change.

The physiological response of detached leaves treated with 20 mM NaHCO_3_ was investigated through measuring chlorophyll, H_2_O_2_, MDA, and ABA contents. These four physiological and biochemical indicators are commonly used to evaluate the leaf senescence process. The chlorophyll content in both the alkaline-stress and dark groups decreased distinctly from days 2 to 6 in comparison to the slight reduction in the control-light group. Moreover, the chlorophyll content was even undetectable in the 20 mM NaHCO_3_ group on day 6 (Figure 1B). As expected, H_2_O_2_ levels in the alkaline-stress group increased progressively from days 2 to 6 during leaf senescence; those in the dark-induced group followed the same trend, steadily increasing from days 2 to 4, and decreasing at day 6, as found in an earlier study (Dong et al., 2021; Figure 1C). MDA content in the alkaline stress and dark groups significantly increased to a maximum on day 4 and then dropped slightly on day 6 compared to the mild increase in the control-light group (Figure 1D). ABA content in both the treatment and control groups peaked on day 4, and then decreased, remaining above the initial value (Figure 1E).

### Transcriptome Sequencing

**FIGURE 2:**
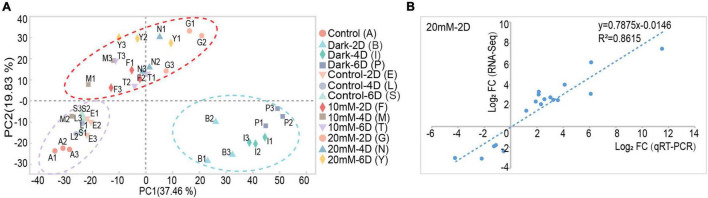
The PCA analysis and qRT-PCR validation of transcriptome data derived from alkali stress-induced detached leaf senescence in *M. truncatula*. **(A)** Principal component analysis (PCA) plot of transcriptome profiles from different conditions. **(B)** Validation of RNA-seq data by qRT-PCR analysis. Correlation of expression changes observed by RNA-seq (*Y*-axis) and qRT-PCR (*X*-axis) relative expression levels from log_2_ fold change of 25 genes in 20 mM NaHCO_3_-light-2 day; data for other groups are shown in Supplementary Figure 2.

The detached leaves were treated under the conditions of control-light, dark, 10 mM, and 20 mM NaHCO_3_ for 0, 2, 4, and 6 days; a total of 13 groups with three biological replicates in each group (in total 39 samples) were sampled for library construction and subsequent sequencing. A total of 283.60 Gb high-quality clean data were obtained. The clean reads from each sample exceeded 7.27 Gb, and the matching to the reference genomic sequence was 81.78–89.05%. The GC content was above 42.60% and the percentage of Q30 bases was at least 92.82% (Supplementary Table 2). Principal component analysis (PCA) showed higher similarity among biological replicates of the same group and higher variability among different groups under different conditions (Figure 2A). The high Pearson correlation values of the biological replicates for the 39 samples achieved the expectation of the experimental design (Supplementary Figure 1). The specific gene expression profiles obtained by qRT-PCR analysis were used for the validation of RNA-Seq data, and the results showed similar expression profiles between RNA-Seq and qRT-PCR analysis (Figure 2B and Supplementary Figure 2).

### Identification of Differentially Expressed Senescence-Associated Genes

**FIGURE 3:**
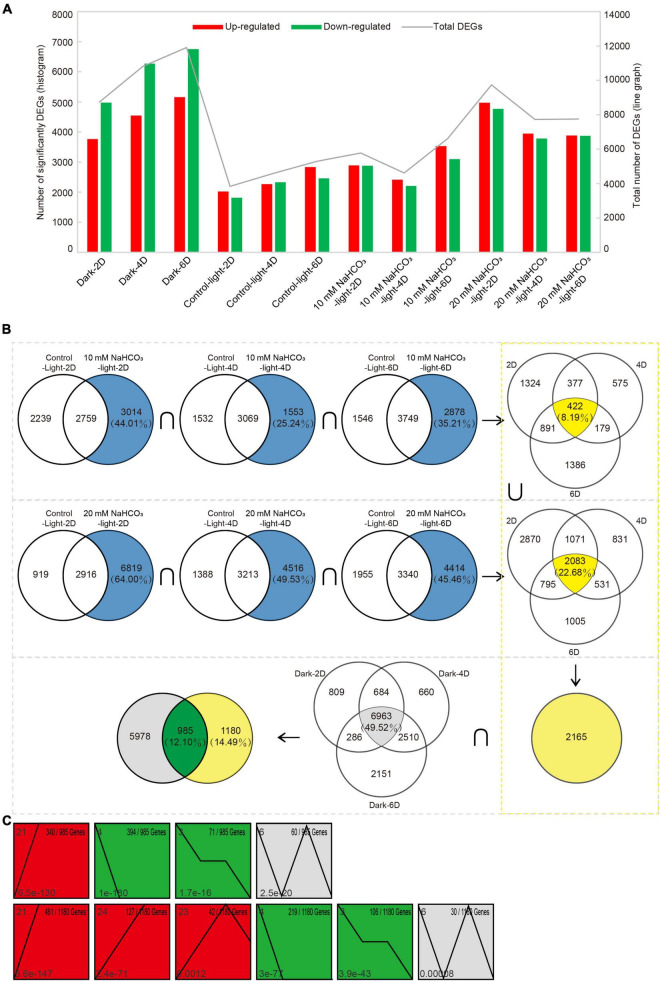
Analysis of differentially expressed senescence-associated genes (SAGs) under alkali stress. **(A)** The number of differentially expressed genes that were upregulated or downregulated during leaf senescence compared with 0 day control prior to treatment, as assessed using the difference analysis software edgeR at thresholds of | log_2_FC| ≥ 1 and *P* < 0.05. **(B)** Venn diagram of DEGs (|log_2_FC| ≥ 1 and *P* < 0.05) in the 10 mM, 20 mM NaHCO_3_ treatments and darkness on day 2, 4, and 6 compared with control-light condition. There were 985 SAGs shared by darkness and NaHCO_3_ treatments across three time points. **(C)** Short time-series expression miner (STEM) analysis of 985 and 1,180 genes across three time points. Each square box indicates a type of expression profile, with the profile order on the upper left and the *P*-value on the bottom left. Only significantly enriched cluster profiles with a *P* < 0.05 threshold are shown.

Compared with day 0 levels, the upregulated and downregulated genes in both the control-dark and 20 mM NaHCO_3_ treatment groups ranged from 8,700 to 11,910, while the number of DEGs in the control-light group was much smaller than that in the treatment group, ranging between 3,800 and 5,300 DEGs (Figure 3A and Supplementary Table 3). The number of upregulated and downregulated genes in the alkaline stress group decreased with extended treatment time, while there was an opposite trend in both control groups.

Venn diagrams at three time points based on all DEGs from both the 10 and 20 mM NaHCO_3_ treatments groups compared with the DEGs from the control-light group were constructed to obtain SAGs in order to identify genes associated with leaf senescence (Figure 3B). We identified 2,165 unique genes in the 10 and 20 mM NaHCO_3_ treatment groups compared with the control group. These genes were then divided into two parts after taking the intersection with the set of SAGs obtained from the dark-induced groups: 985 SAGs and 1,180 genes (Supplementary Table 4). STEM temporal pattern analysis showed genes with the same expression type were grouped into the same profile (Figure 3C). The 985 SAGs were divided into four significant gene expression profiles (*P* < 0.05), including one upregulated profile (red; profile 21; 340 genes), two downregulated profiles (green; profiles 3 and 4; 71 and 394 genes), and one other profile (gray; profile 6; 60 genes). The 1,180 genes were divided into six significant gene expression profiles: three being upregulated (profiles 21, 23, and 24; with 461, 42, and 127 genes, respectively), two downregulated (profiles 3 and 4; with 106 and 219 genes, respectively), and one other profile (profile 6; 30 genes).

### Gene Ontology and Kyoto Encyclopedia of Genes and Genomes Pathway Enrichment Analysis

GO enrichment analysis characterizes the gene function and relations in three categories: biological processes (BP), molecular functions (MF), and cellular components (CC). As shown in Table 1, the GO terms with the top 10 highest enrichment degree were all went to BP category for 985 SAGs, while for 1,180 DEGs the top 10 highest enrichments were classified into CC and BP categories. For the 985 SAGs, the top GO terms were mainly involved in “small molecule catabolic and metabolic process,” “cellular amino acid metabolic processes,” “carboxylic acid catabolic and metabolic process,” and “organic acid and substance catabolic process” of BP category; while among the 1,180 DEGs, the top GO terms in BP category were “aerobic respiration,” “aerobic electron transport chain,” “ion transport,” and “cation transport”; also in CC category, “mitochondrion,” “oxidoreductase complex,” “NADH dehydrogenase complex,” and “Membrane-bounded organelle” were significantly enriched. In all, the enrichment of GO terms with higher degrees from the two gene sets (985 SAGs and 1,180 DEGs) was remarkably significantly different, indicating that the genes of the two gene sets play different roles under alkaline stress.

**TABLE 1:** Gene ontology (GO) and Kyoto Encyclopedia of genes and genomes (KEGG) pathways enrichment analysis of DEGs (Figure 3B, 985 SAGs and 1,180 genes) shared by darkness and alkaline-induced leaf senescence at three time points.

From	No.	GO/KEGG pathway id	GO/KEGG pathway term	Category	Sample gene number			Background gene number	Rich factor	P-adjust
					
					Total	Up	Down			
985 SAGs	1	GO:0010027	Thylakoid membrane organization	Biological process	17	0	17	104	0.16346	0.00076
	2	GO:0044282	Small molecule catabolic process	Biological process	23	18	5	224	0.10268	0.00076
	3	GO:0006520	Cellular amino acid metabolic process	Biological process	32	15	17	569	0.05624	0.00076
	4	GO:0019752	Carboxylic acid metabolic process	Biological process	51	28	23	1160	0.04397	0.00076
	5	GO:0016054	Organic acid catabolic process	Biological process	14	12	2	150	0.09333	0.00076
	6	GO:0046395	Carboxylic acid catabolic process	Biological process	14	12	2	150	0.09333	0.00076
	7	GO:1901575	Organic substance catabolic process	Biological process	68	42	26	1670	0.04072	0.00076
	8	GO:0044281	Small molecule metabolic process	Biological process	82	39	43	2000	0.04100	0.00076
	9	GO:0019252	Starch biosynthetic process	Biological process	10	2	8	75	0.13333	0.00076
	10	GO:0043436	Oxoacid metabolic process	Biological process	54	28	26	1248	0.04327	0.00076
	1	GO:0005739	Mitochondrion	Cellular component	49	42	7	747	0.06560	0.00107
1180 Genes	2	GO:1990204	Oxidoreductase complex	Cellular component	12	12	0	92	0.13043	0.00107
	3	GO:0009060	Aerobic respiration	Biological process	7	7	0	29	0.24138	0.00127
	4	GO:0030964	NADH dehydrogenase complex	Cellular component	7	7	0	31	0.22581	0.00160
	5	GO:0019646	Aerobic electron transport chain	Biological process	5	5	0	13	0.38462	0.00185
	6	GO:0006811	Ion transport	Biological process	51	37	14	1136	0.04489	0.00315
	7	GO:0006812	Cation transport	Biological process	35	24	11	690	0.05072	0.00616
	8	GO:0043227	Membrane-bounded organelle	Cellular component	236	156	80	7800	0.03026	0.00746
	9	GO:0055085	Transmembrane transport	Biological process	64	52	12	1608	0.03980	0.01016
	10	GO:1902600	Proton transmembrane transport	Biological process	15	14	1	227	0.06608	0.04282
	1	Map00630	Glyoxylate and dicarboxylate metabolism	Carbohydrate metabolism	10	4	6	112	0.08929	0.029559
985 SAGs	2	Map00280	Valine, leucine and isoleucine degradation	Amino acid metabolism	8	6	2	68	0.11765	0.033328
	3	Map00350	Tyrosine metabolism	Amino acid metabolism	7	6	1	65	0.10769	0.033824
	4	Map00330	Arginine and proline metabolism	Amino acid metabolism	9	5	4	103	0.08738	0.043300
1180 Genes	1	Map04714	Thermogenesis	Environmental adaptation	22	21	1	256	0.08594	0.00056
	2	Map00190	Oxidative phosphorylation	Energy metabolism	22	22	0	291	0.075601	0.00207

KEGG enrichment analysis showed that the most significantly enriched pathways in 985 genes were related to “glyoxylate and dicarboxylate metabolism,” “valine, leucine, and isoleucine degradation,” “tyrosine metabolism,” and “arginine and proline metabolism.” On the contrary, thermogenesis, and oxidative phosphorylation were the enriched KEGG pathway terms for the 1,180 genes.

**FIGURE 4:**
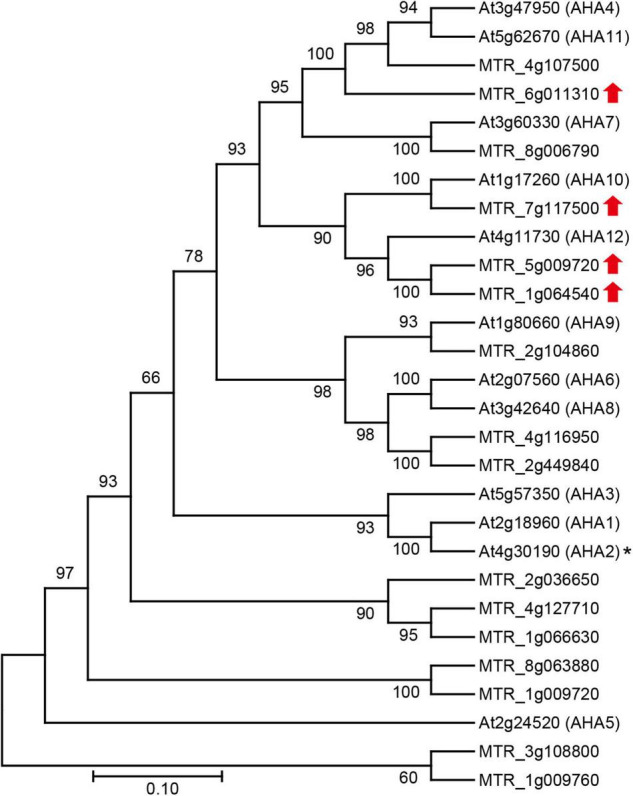
Phylogenetic analysis of PM H^+^-ATPase gene family members in *M. truncatula* and *Arabidopsis thaliana*. The upward red arrows indicates that the genes are up-regulated in the alkali stress-induced detached leaf senescence in *M. truncatula*; and the asterisk shows the alkaline-stress-regulated PM H^+^-ATPase gene in Arabidopsis. The scale bar indicates the sequence divergence is 0.10 per unit bar.

It is reported that regulation of plasma membrane (PM) H^+^-ATPase activity is important for plant adaptation to alkali stress and enhancement of higher leaf photosynthesis (Fuglsang et al., 2007; Yang et al., 2010, 2019; Zhang M. et al., 2021). Four key genes related to PM H^+^-ATPase are up-regulated expression, and phylogenetic analysis indicated that MTR_5g009720, MTR_6g011310, MTR_7g117500, and MTR_1g064540 are orthologs of Arabidopsis PM H^+^-ATPase (Figure 4).

### Transcription Factor Analysis

TFs play an important role in regulating leaf senescence; the PlantTFDB 4.0 match analysis was used for predicting TFs. We identified 101 and 173 TFs in 985 SAGs and 1,180 DEGs, belonging to 16 and 26 TF families, respectively (Supplementary Table 5). The most typical representative TF families in 985 SAGs included *bHLH* (seven genes), *MYB* (four genes), and *WRKY* (three genes), while *B3* (seven genes), *MYB* (seven genes), and *HB-other* (six genes) were the most representatives in 1,180 DEGs (Table 2). As shown in Table 2 for the representatives from 1,180 DEGs, most *B3*, *HB-other*, and *ARF* TFs were downregulated, whereas the *MYB* and *WRKY* TF families were upregulated.

**TABLE 2:** Transcription factors (TFs) predicted from 985 SAGs and 1,180 DEGs (Figure 3B).

No.	TF family	985 SAGs Gene number	Up	Down	TF family	1180 DEGs Gene number	Up	Down
1	bHLH	7	3	4	B3	7	1	6
2	MYB	4	2	2	MYB	7	6	1
3	WRKY	3	1	2	HB-other	6	0	6
4	CO-like	3	0	3	ARF	5	0	5
5	Dof	3	2	1	GRAS	5	2	3
6	DBB	2	0	2	WRKY	5	4	1
7	MYB-related	2	2	0	ERF	3	3	0
8	NAC	2	2	0	MIKC	3	2	1
9	bZIP	2	2	0	M-type	3	2	1
10	ERF	2	0	2	bHLH	3	1	2
								

The families and numbers of TFs have big differences in the two gene sets. The *NAC* and *bZIP* TF families belong to 985 SAGs, and these TF families are widely reported in the regulation of senescence. The *B3*, *HB-other*, and *ARF* TF families in the 1,180 DEGs play an important role in abiotic stress defense responses. The *MYB*, *WRKY*, and *bHLH* families are multifunctional but essentially regulate plant senescence directly or indirectly.

### Time-Course Senescence-Associated Gene Analysis

**FIGURE 5:**
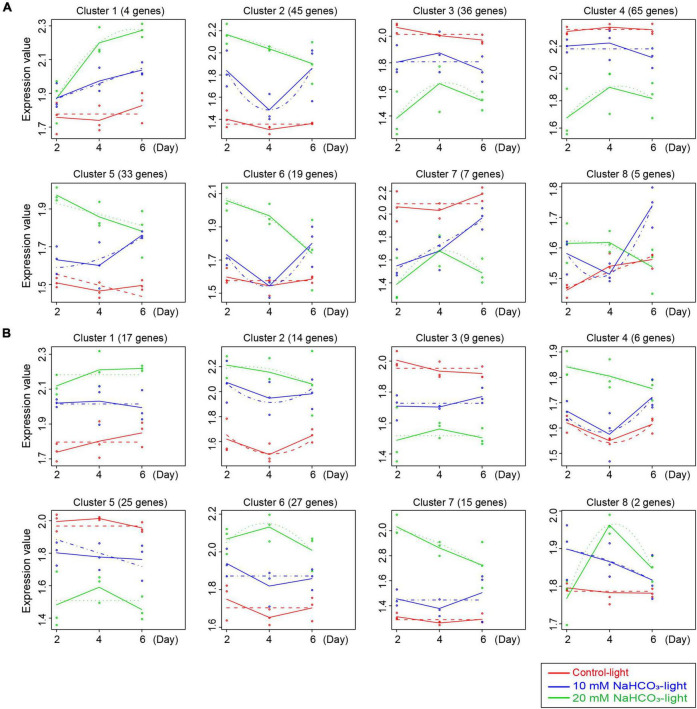
Data visualization according to the cluster analysis. Each map shows the average expression profiles of the gene clusters from all samples. The horizontal coordinates represent different time points, and the vertical coordinates represent the treated expression values. Different colored lines represent different groups (red, blue, and green lines represent the control light, 10 mM NaHCO_3_-light, and 20 mM NaHCO_3_-light groups, respectively) and show the actual average value of gene expression at each time point. Different points represent the actual average expression levels for specific individual samples. The dotted lines represent the fitted curves of gene expression at each time point. **(A,B)** Are the cluster analysis for 985 SAGs and 1,180 DEGs, respectively.

Time-course gene expression analysis found that 985 SAGs and 1,180 DEGs (Figure 3B) were divided into eight clusters, each of which included certain genes with the same expression patterns. The gene expression trend differences between the control and treated groups are illustrated in Figure 5. Among the 985 SAGs, the 106 genes were upregulated in clusters 1, 2, 5, 6, and 8, and the 108 downregulated genes (50.5%) were enriched in clusters 3, 4, and 7 (Figure 5A). In clusters 1, 2, 4, 6, 7, and 8 of 1,180 DEGs, 81 genes were upregulated, while in clusters 3 and 5, 34 genes (29.6%) were downregulated (Figure 5B). The expression patterns of all genes are shown in Supplementary Figure 3.

To elucidate the expression pattern and function of genes in different clusters of 985 SAGs and 1,180 DEGs, we performed KEGG pathway enrichment analysis. Among the 985 SAGs, the genes related to nutrient cycling are enriched in the clusters of upregulated genes, including clusters 1, 2, and 8 with strikingly expressed amino acid metabolism-related genes. The genes related to photosynthesis are enriched in clusters 3, 4, and 7 with downregulated expression patterns. There was a strong difference of enriched genes’ function between 985 SAGs and 1,180 DEGs. Among the 1,180 DEGs, the genes related to oxidative phosphorylation and thermogenesis are enriched in clusters of upregulated genes, especially in clusters 1 and 5. It is worth noting that there are arginine and proline metabolism-related genes present in clusters 7 and 8, and these genes are acting to relieve osmotic stress (Supplementary Table 6).

### Integration Analysis of the Physiological Data and Transcriptome

Physiological indicators were closely linked to the transcriptome during plant leaf senescence (Table 3). A large number of genes related to nutrient metabolism were differentially expressed, such as 59 DEGs involved in amino acid metabolism, which could be expected to rapidly loose and shift nutrients during leaf senescence; and 76 chloroplast and thylakoid metabolism-related genes were observed, consistent with leaf yellowing and chlorophyll breakdown. Alkaline stress induced ROS production and accelerated leaf senescence; 178 genes related to oxidation activity were differentially expressed, in alignment with the increased H_2_O_2_ and MDA contents observed. Furthermore, the accumulation of ROS increased the permeability of the cell membrane in leaves, causing 169 genes related to ion transport to be differentially expressed (Supplementary Table 7).

**TABLE 3:** Gene ontology (GO) enrichment analysis of DEGs (Figure 3B, 2,165 genes) that regulation of physiological activity.

Function	No.	GO id	GO term	Category	Sample gene number	Background gene number	Rich factor	P-adjust
Oxidation activity	1	GO:0055114	Oxidation-reduction process	Biological process	178	2739	0.06499	0.00069
	2	GO:0015980	Energy derivation by oxidation of organic compounds	Biological process	11	61	0.18033	0.00382
	3	GO:0034440	Lipid oxidation	Biological process	8	40	0.20000	0.01422
	4	GO:0019395	Fatty acid oxidation	Biological process	7	35	0.20000	0.02858
Amino acid metabolism	1	GO:1901605	Alpha-amino acid metabolic process	Biological process	41	364	0.11264	0.00069
	2	GO:0006520	Cellular amino acid metabolic process	Biological process	59	569	0.10369	0.00069
	3	GO:0009064	Glutamine family amino acid metabolic process	Biological process	17	106	0.16038	0.00069
	4	GO:1901606	Alpha-amino acid catabolic process	Biological process	13	84	0.15476	0.00435
	5	GO:0008652	Cellular amino acid biosynthetic process	Biological process	26	259	0.10039	0.00516
	6	GO:1901607	Alpha-amino acid biosynthetic process	Biological process	24	230	0.10435	0.00641
	7	GO:0009063	Cellular amino acid catabolic process	Biological process	13	89	0.14607	0.00704
	8	GO:0003333	Amino acid transmembrane transport	Biological process	10	73	0.13699	0.04495
Chloroplast and thylakoid metabolism	1	GO:0044434	Chloroplast part	Cellular component	87	1114	0.07810	0.00069
	2	GO:0009570	Chloroplast stroma	Cellular component	36	399	0.09023	0.00378
	3	GO:0009507	Chloroplast	Cellular component	76	1139	0.06673	0.01457
	4	GO:0010027	Thylakoid membrane organization	Biological process	17	104	0.16346	0.00069

**FIGURE 6:**
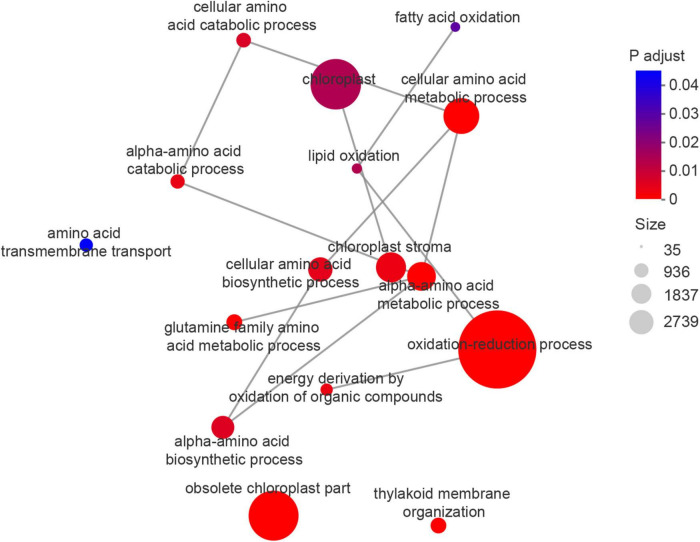
Enrichment network map for GO terms (Table 3), highlighting the relationship between GO terms and GO terms, GO terms and genes.

The enrichment network map for GO terms described in Table 3 is displayed in Figure 6, and highlights the relationships between GO terms and GO terms, and between GO terms and genes. Functionally related GO terms were highly correlated.

### Responses of Detached Leaves to Salt Stress and Alkali Stress

**FIGURE 7:**
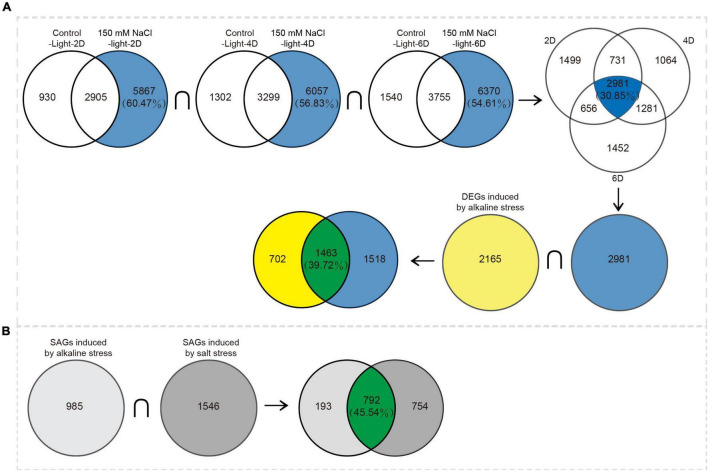
Venn diagram analysis of DEGs induced by alkaline stress and by salt stress. **(A)** Differences between DEGs induced by alkaline stress and by salt stress. **(B)** Differences between SAGs induced by alkaline stress and by salt stress.

The transcription profiles of salt stress have been analyzed in our previous study (Dong et al., 2021). Combined with the previous data, analysis showed that 1,463 DEGs were induced by both salt and alkali stresses, and these genes were mostly associated with nutrient cycling metabolism. The 702 DEGs were induced by alkali stress and enriched in thermogenic and oxidative phosphorylation pathways; 1,518 DEGs were induced by salt stress and enriched in porphyrin and chlorophyll metabolism, the citrate cycle (TCA cycle), and photosynthesis (Figure 7A and Supplementary Table 8). On this basis, we focused on the differential expression of senescence-associated genes in the two stresses; 792 SAGs were induced under both salt and alkali stresses (Figure 7B).

## Discussion

We obtained high-precision RNA-seq data from highly controlled and detached leaves from individual *M. truncatula* plants at four time points; and analyzed data through standard procedures. In addition, we compared the data from this study (alkali stress-induced leaf senescence) with data from a previous study (salt stress-induced leaf senescence) to construct a complete and rigorous experimental design strategy.

**FIGURE 8:**
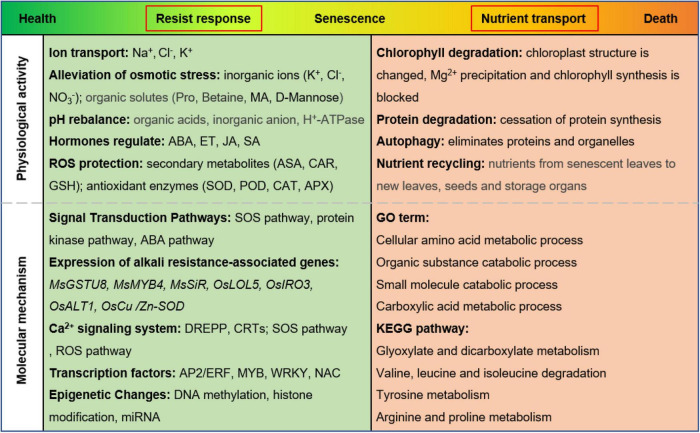
A model of leaf senescence regulation under alkaline stress in *M. truncatula.*

Salinity and alkaline conditions are widely recognized as abiotic stresses which restrict crop production as well as ecological environment construction. High salinity can affect the growth and development of plants by accelerating leaf senescence and plant death (Guo et al., 2021). In this process, chlorophyll degrades, and leaf color changes from green to yellow (Xue et al., 2021). Our physiological data confirmed this phenomenon (Figures 1A,B). We found that alkaline stress was more toxic than salt stress because of more rapid chlorophyll breakdown and more severely eroded leaves. Transcriptome data also showed that the molecular mechanisms of leaf senescence induced by the two stresses were not the same (Figure 7 and Supplementary Table 8), and many studies have reached similar conclusions (Guo et al., 2009; Yang et al., 2009; Li et al., 2010). Salt stress (mainly NaCl) and alkali stress (mainly Na_2_CO_3_ and NaHCO_3_) can accelerate leaf senescence through ion toxicity, osmotic stress, and oxidative stress. However, alkali stress causes more damage to leaves in less time, and the molecular mechanism is more complex (Guo et al., 2015). We propose a model of the physiological activities and molecular mechanisms of *M. truncatula* leaf responding to alkali stress. As shown in Figure 8, in the early stage of leaves upon to alkali stress, plant mainly faces to resist the stress. The corresponding physiological activities included ion transport, alleviation of osmotic stress, pH rebalance, hormone regulation, ROS protection; In molecular mechanisms, more changes focus on: (1) signal Transduction Pathways; (2) expression of alkali resistance-associated genes; (3) Ca^2+^ signaling system; (4) transcription factors; (5) epigenetic Changes. In the late stage of leaves under alkali stress, plant mainly shift to nutrient transport. The corresponding physiological activities included chlorophyll degradation, protein degradation and nutrient recycling. This strategy adjustment means that plant is about to give up the leaves that suffer from alkali stress.

Alkaline stress greatly disrupts the ionic dynamic balance in plants and interferes with the uptake of mineral elements, leading to excessive accumulation of Na^+^ in the leaf cytoplasm, causing ion toxicity which damages cell organelles (Yang et al., 2008; Ruiz et al., 2016). In our assay, enrichment of DEGs related to ion transport and transmembrane transport was detected, with most being upregulated (Table 1). It has been reported that alkali stress can affect the distribution of ions in plant organs; for example, a large amount of Na^+^ and Cl^–^ accumulates in old leaves, and the content of Na^+^ and Cl^–^ in new leaves is lower (Foolad, 2004; Wang et al., 2012). The Na^+^ content in the leaves also increases significantly with increasing alkali stress (Dai et al., 2014; Abdel Latef and Tran, 2016). Plants maintain ion balance through ion metabolism, an important mechanism for adaptation to alkali stress (Blumwald et al., 2000).

Alkali stress can lead to massive water shortages in the leaves and accelerate leaf senescence (Zhang P. et al., 2021). In our study, genes related to metabolic intermediates, such as organic acid catabolic process and proline metabolism, were found to be significantly enriched; these metabolic intermediates act to relieve osmotic stress and contribute to the maintenance of normal water content in the leaves (Ali et al., 2008).

The effect of oxidative damage on leaf senescence is notable. High salinity can induce superoxide radicals, resulting in membrane lipid peroxidation and disruption of membrane integrity, which is a direct and important cause of leaf senescence. MDA is one of the main products of membrane lipid peroxidation (Bao et al., 2009), and we found that MDA content accumulated during leaf senescence (Figure 1D), suggesting that leaves suffered oxidative damage under alkaline stress. Similar results have been reported elsewhere (Zou et al., 2020; Wei et al., 2021; Wu et al., 2021). H_2_O_2_ is a common ROS involved in plant senescence. It can oxidize macromolecules and damage cell membranes, causing senescence (Halliwell, 1984; Chen et al., 2012). The H_2_O_2_ content increased during alkaline-induced senescence (Figure 1C). Transcriptional profile analysis also confirmed this result, with a large number of DEGs enriched in the oxidation-reduction process, energy derivation by oxidation of organic compounds, lipid oxidation, and fatty acid oxidation (Table 3). The levels of MDA and H_2_O_2_, as markers of oxidative stress-induced cellular damage, are widely used to assess the degree of plant senescence in *Arabidopsis* (Cui et al., 2013), alfalfa (Wei et al., 2021), rice (Chen et al., 2018), and adzuki bean (Li et al., 2020). Plants have ROS scavenging systems, including secondary metabolites and antioxidant enzymes, to reduce accumulated ROS and defend against oxidative damage (Gong et al., 2013; Wu et al., 2021). Secondary metabolites act as antioxidants to help plants scavenge ROS, such as ASA, carotenoids, glutathione, and certain low-molecular-weight compounds (Fan et al., 2021; Fang et al., 2021). Our transcriptome analysis showed that DEGs were enriched in KEGG pathways of secondary metabolites, such as carotenoid biosynthesis and glutathione metabolism (Supplementary Figure 4 and Supplementary Table 9). These DEGs may be involved in ROS elimination. Antioxidant enzymes play the most important role in the ROS scavenging system, and mainly include superoxide dismutase, peroxidase, and catalase, which catalyze H_2_O_2_ into O_2_ and H_2_O (Sun et al., 2019), and ascorbic acid peroxidase, which reduces membrane lipid peroxidation by scavenging MDA (Fang et al., 2021). It has been reported that overexpression of Cu/Zn-superoxide dismutase can increase the degree of tolerance to H_2_O_2_, thus reducing the damage caused by alkaline stress in plants (Wu et al., 2016). Additionally, antioxidant enzymes and antioxidants work together to effectively scavenge ROS and alleviate oxidative stress (Fu et al., 2017). Although plants have developed a series of regulatory adaptive mechanisms to resist alkali stress-induced senescence, the regulatory adaptive mechanisms of plants lose their effect if the concentration of alkali stress exceeds a threshold, leading to senescence and death.

In our assay, 2,165 DEGs were identified through transcriptomics of alkali-induced detached leaves. For further analysis, we divided the 2,165 DEGs into two parts: 985 SAGs and 1,180 DEGs; 985 genes were identical to the SAGs of the dark treatment group, which we believe are directly involved in the leaf senescence process. The remaining 1,180 genes may indirectly regulate leaf senescence (Figure 3B). GO and KEGG pathway enrichment analysis confirmed our conjecture that the genes of the two gene sets play different roles in leaf senescence: 985 SAGs were mainly enriched in nutrient cycling processes, such as cellular amino acid metabolic processes and organic substance catabolic processes, indicating nutrient redistribution. The 1,180 SAGs were significantly enriched in oxidoreductase complex, aerobic respiration, and ion transport (Table 1), most of which were upregulated during senescence (Figure 5).

TFs are the most significant components in the regulation of leaf senescence (Guo et al., 2004; Balazadeh et al., 2008). In our study, we identified 101 and 173 TFs among the 985 SAGs and 1,180 DEGs, respectively. The *MYB*, *WRKY*, *NAC*, and *bZIP* TF families belonged to the 985 SAGs, which are known to be involved in the regulation of senescence (Guo et al., 2004; Ay et al., 2010; Guo and Gan, 2011). The *B3*, *HB-other*, and *ARF* TF families in the 1,180 DEGs play an important role in abiotic stress defense responses. *MYB*, *WRKY*, and *bHLH* are multifunctional but essentially regulate plant senescence directly or indirectly.

The leaf is an important site for photosynthesis in plants and is sensitive to senescence. Premature leaf senescence caused by numerous environmental stresses (such as darkness, drought, salt, and alkali stress) can result in significant yield loss and quality reduction, especially in plants that focus on harvesting leaves, such as alfalfa. Therefore, the regulation of appropriate senescence time under abiotic stress needs investigation, with the aim to obtain varieties with an ideal senescence time. Alfalfa is considered a major forage crop worldwide, due to its high yield, high nutrient quality, and wide range of adaptation; however, premature leaf senescence caused by stress affects the quality and yield of alfalfa.

In summary, this study described detailed expression profiles of leaf senescence induced by alkali stress in *M. truncatula*, and annotated many SAGs. New candidate genes have been identified for further senescence resistance breeding, in order to improve the biomass and quality of forage crops under alkaline stress.

## Data Availability Statement

The datasets presented in this study can be found in online repositories. The names of the repository/repositories and accession number(s) can be found below: National Center for Biotechnology Information (NCBI) BioProject database under accession number PRJNA805030.

## Author Contributions

HX, Z-YW, and SD conceived and designed the experiments. SD, WP, HL, and KZ performed the experiments. SD, ZL, LC, and GY analyzed the data. SD wrote the manuscript. HX and Z-YW revised the manuscript. All authors read, revised and approved the final manuscript.

## Conflict of Interest

The authors declare that the research was conducted in the absence of any commercial or financial relationships that could be construed as a potential conflict of interest.

## Publisher’s Note

All claims expressed in this article are solely those of the authors and do not necessarily represent those of their affiliated organizations, or those of the publisher, the editors and the reviewers. Any product that may be evaluated in this article, or claim that may be made by its manufacturer, is not guaranteed or endorsed by the publisher.

## Footnotes

1
https://github.com/OpenGene/fastp

2
http://ccb.jhu.edu/software/hisat2/index.shtml

3
https://ccb.jhu.edu/software/stringtie/

4
http://deweylab.biostat.wisc.edu/rsem/

5
http://bioconductor.org/packages/stats/bioc/edgeR/

6
http://www.geneontology.org/

7
https://github.com/tanghaibao/Goatools

8
http://kobas.cbi.pku.edu.cn/home.do

9
http://www.genome.jp/kegg/

10
http://planttfdb.cbi.pku.edu.cn/

11
http://www.bioconductor.org/packages/release/bioc/html/maSigPro.html

## Supplementary Material

Supplementary Figure 1 Correlation analysis showed the direct association between samples in the same treatment.

Supplementary Figure 2 Validation of RNA-seq data by the qRT-PCR analysis. Correlation of expression changes observed by RNA-seq (*Y*-axis) and qRT-PCR (*X*-axis) relative expression levels from log_2_ Fold Change of 25 genes in different groups.

Supplementary Figure 3 Data visualization according to cluster analysis. The horizontal coordinates represent specific samples, the vertical coordinates represent the expression values after treatment log_10_(x + 1) and the different colored lines represent the trend of different genes in all samples.

Supplementary Figure 4 Pathview analysis of the Carotenoid (CAR) biosynthesis and Glutathione (GSH) metabolism pathway. The genes in the red rectangle are up-regulated.

Supplementary Table 1 Primers of genes used for real-time quantitative PCR in this study.

Supplementary Table 2 Project sample information, raw data, quality control data and comparison data.

Supplementary Table 3 Differentially expressed genes (DEGs) at days 2, 4, and 6.

Supplementary Table 4 985 SAGs and 1,180 genes are obtained using the Venn diagram.

Supplementary Table 5 TF family analysis data.

Supplementary Table 6 KEGG pathway enrichment analysis in individual cluster.

Supplementary Table 7 169 DEGs related to ion transport.

Supplementary Table 8 Responses of detached leaves to salt stress and alkali stress.

Supplementary Table 9 Carotenoid biosynthesis and glutathione metabolism.
