# Supplementary figures and images for: Transcriptome Analysis of Leaf Senescence Regulation Under Alkaline Stress in Medicago truncatula
Source: Front Plant Sci. 2022 Apr 28;13:881456. doi: 10.3389/fpls.2022.881456 (PMC9096890; doi:10.3389/fpls.2022.881456)

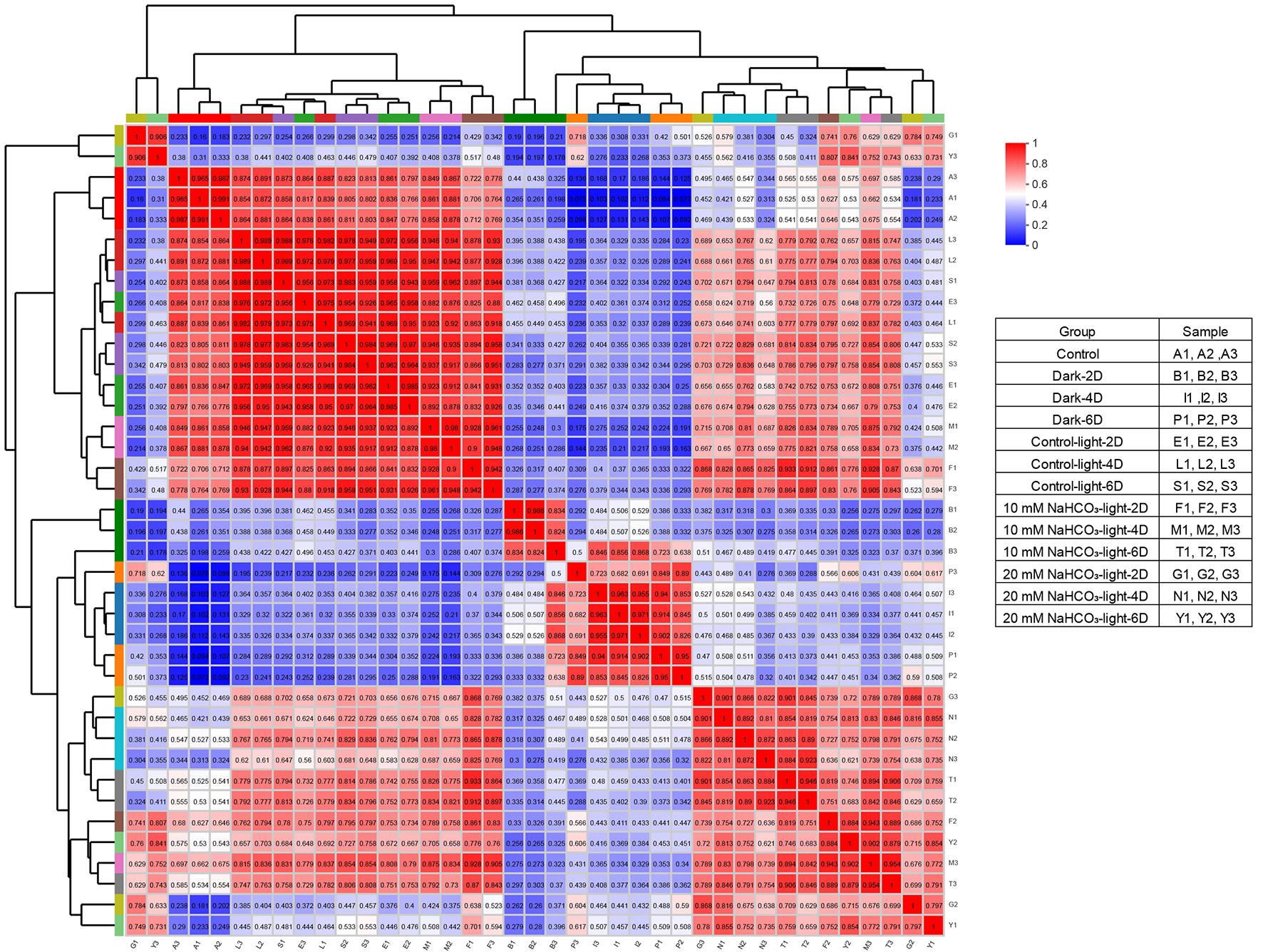

Supplement: Supplementary Figure 1 — Correlation analysis showed the direct association between samples in the same treatment. [file Image_1.TIF]

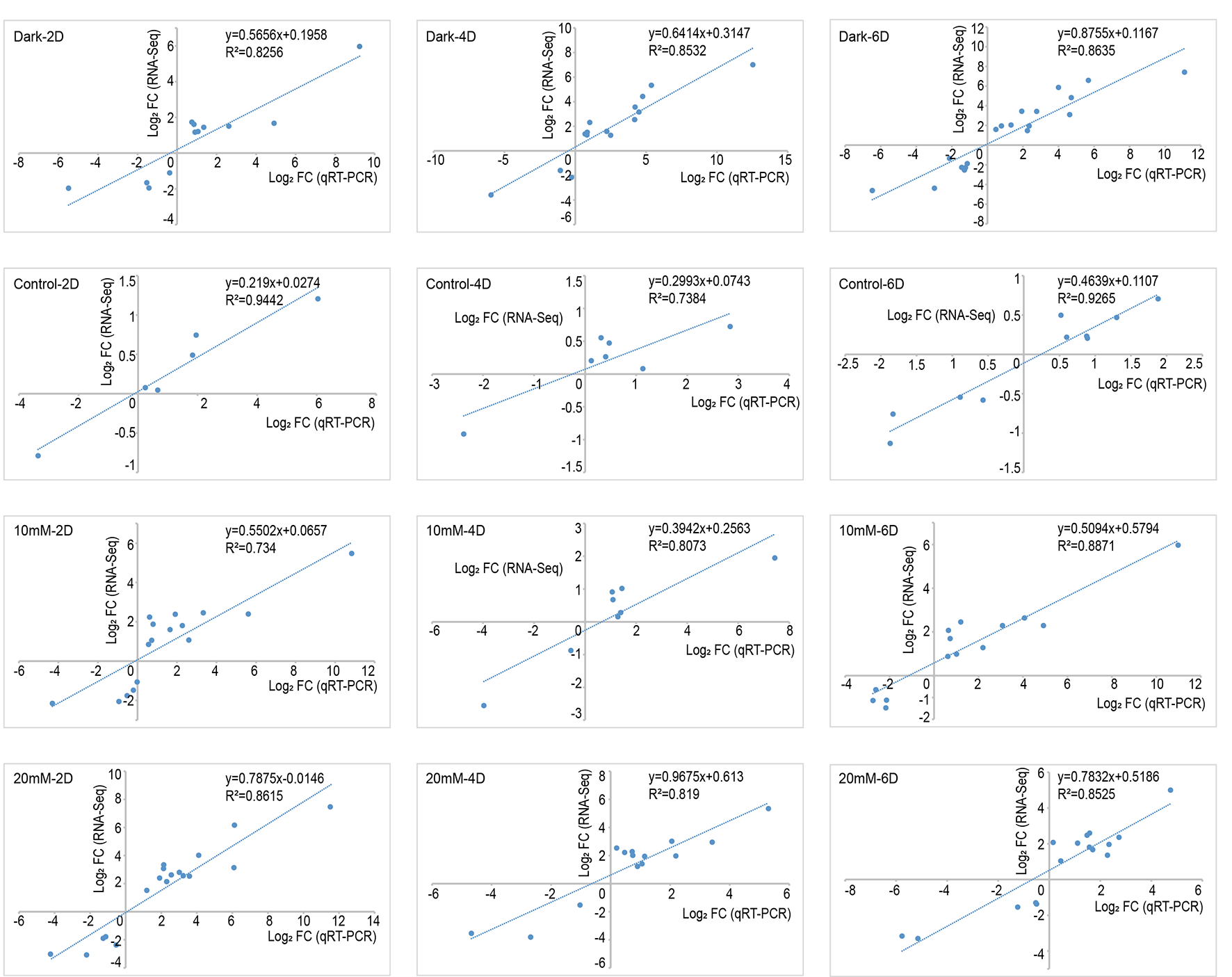

Supplement: Supplementary Figure 2 — Validation of RNA-seq data by the qRT-PCR analysis. Correlation of expression changes observed by RNA-seq (Y-axis) and qRT-PCR (X-axis) relative expression levels from log2 Fold Change of 25 genes in different groups. [file Image_2.TIF]

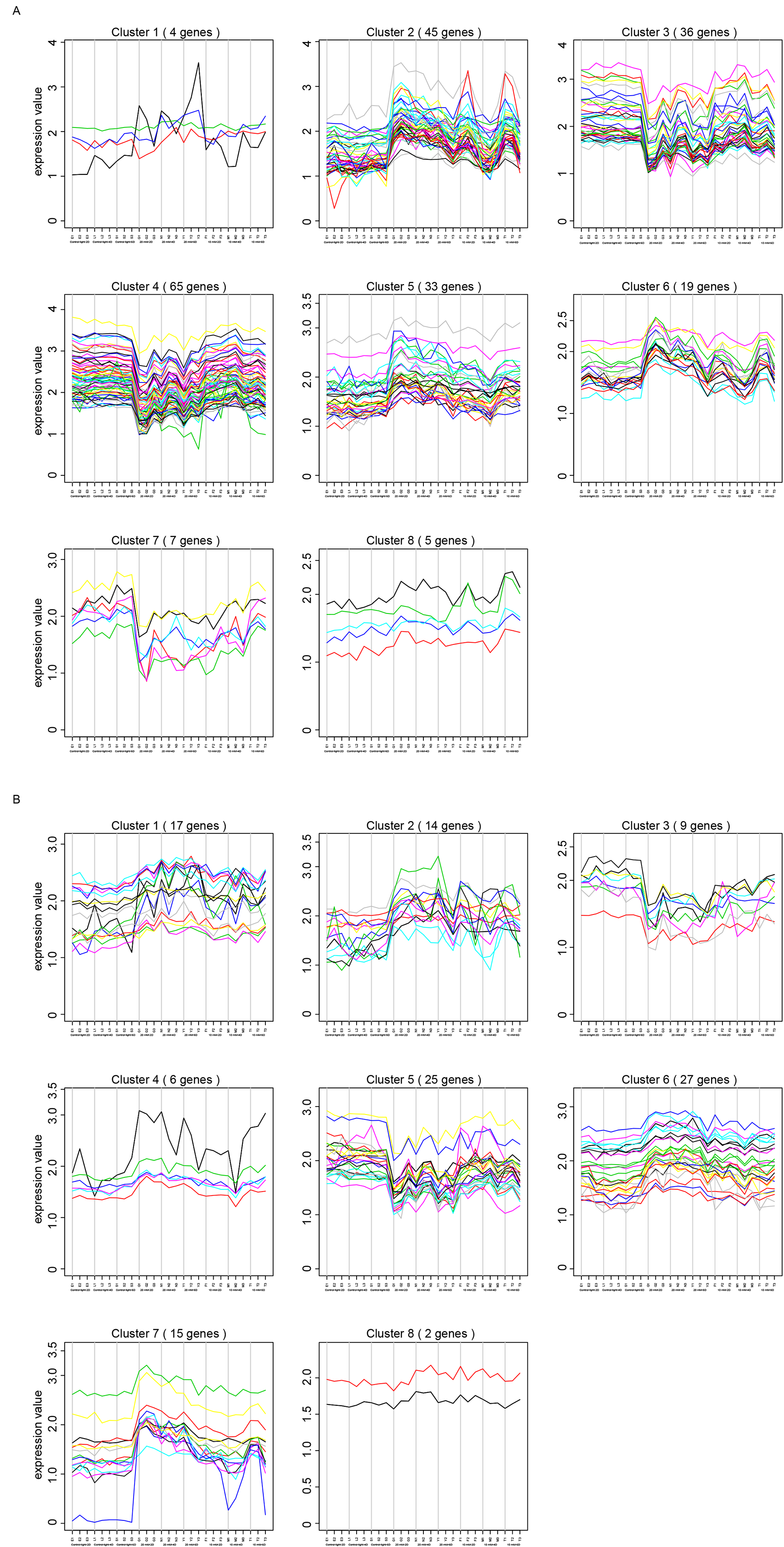

Supplement: Supplementary Figure 3 — Data visualization according to cluster analysis. The horizontal coordinates represent specific samples, the vertical coordinates represent the expression values after treatment log10(x + 1) and the different colored lines represent the trend of different genes in all samples. [file Image_3.TIF]

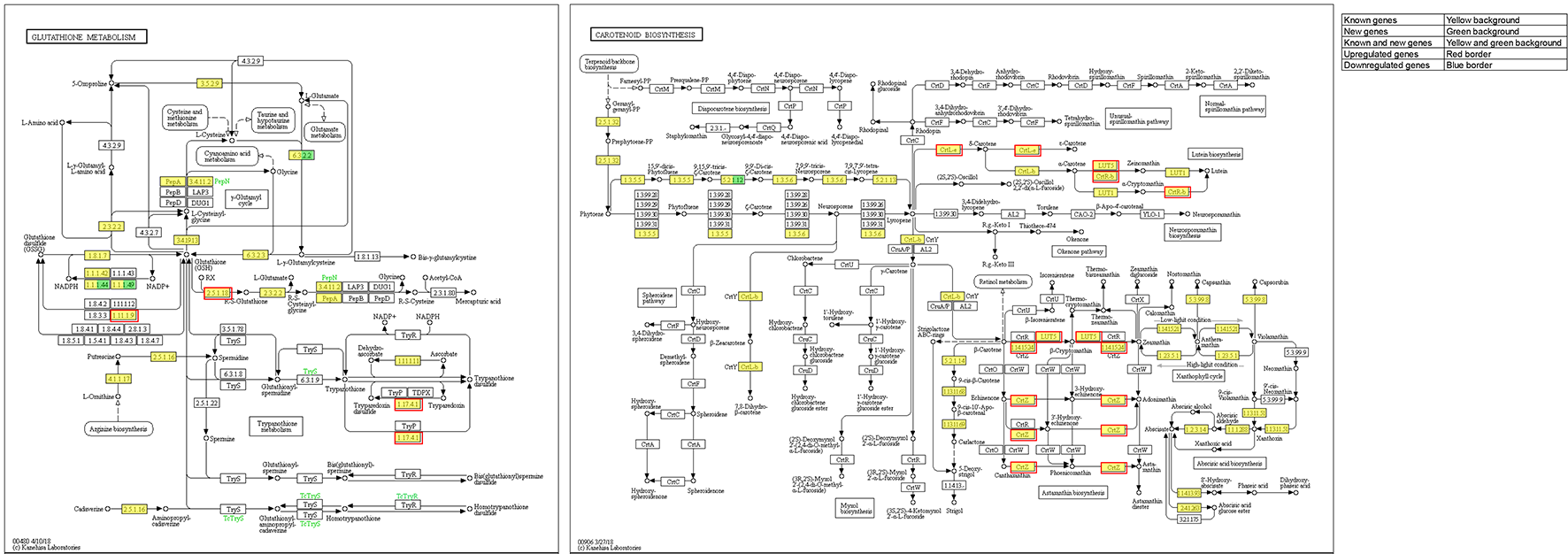

Supplement: Supplementary Figure 4 — Pathview analysis of the Carotenoid (CAR) biosynthesis and Glutathione (GSH) metabolism pathway. The genes in the red rectangle are up-regulated. [file Image_4.TIF]
